# Efficiency of tourism development in China’s major cities under the constraint of PM2.5

**DOI:** 10.1371/journal.pone.0255508

**Published:** 2021-08-11

**Authors:** Jiajia Li, Kaifeng Duan, Quanwei Xu, Xuefei Sun, Yanwei Zhang, Changhua Hua

**Affiliations:** 1 Hospitality Management School, Shanghai Business School, Shanghai, China; 2 School of Economics and Management, Tongji University, Shanghai, China; 3 College of Public Administration, Huazhong University of Science and Technology, Wuhan, China; 4 School of Business Administration, Shanghai Lixin University of Accounting and Finance, Shanghai, China; Northeastern University (Shenyang China), CHINA

## Abstract

Climate / weather factors are important factors for tourists to choose tourist destinations. With the public’s attention to the influence of haze, air quality will have a profound impact on the development of tourism in tourist destinations. Based on the Epsilon-based Measure (EBM) super-efficiency model and Global Malmquist–Luenberger index analysis method, this paper aims to study the tourism development efficiency of 58 major cities in China from 2001 to 2016 and analyse the total factor productivity in the development of urban tourism and the changing driving factors in consideration of the undesirable output of haze characterised by PM2.5 emission concentration. The study findings show that the overall efficiency of tourism development of 58 cities is not high in 2001–2016, but the tourism development efficiency of all cities is increasing year by year. Under the constraint of haze, the efficiency of urban tourism development is not directly proportional to the degree of urban development. The overall redundancy rate of each input index is slightly high, and the redundancy of PM2.5 emission concentration has a considerable effect on the efficiency of urban tourism development. The overall change trend in total factor productivity in the development of urban tourism is improved, mainly due to the improvement of technological progress factors. On this basis, the corresponding policy implications are concluded according to high-efficiency and high-quality development of tourism in 58 major cities.

## 1 Introduction

In the more than four decades from the 1978 implementation of reform and opening up to 2019, China’s urbanisation rate has increased from 10.6% to 60.6% (*Data source*: *National Bureau of Statistics of China*. *China Statistical Yearbook [M]*.*Beijing*: *China Statistics Press*, *2020*). Urbanisation has become an important way to support China’s economic growth [1]. With the rapid development of urbanisation, the development of China’s tourism industry also presents a rapid growth trend. *Basic Situation of Tourism Market in 2018* released by China Tourism Academy in February 2019 reports that the comprehensive contribution of China’s tourism industry to GDP in 2018 is 9.94 trillion CNY, accounting for 11.04% of the total GDP. A total of 79.91 million people are directly and indirectly employed in the tourism industry, accounting for 10.29% of the total employed population in China. *Guiding Opinions of General Office of the State Council on Promoting the All-round Tourism Development in 2018* suggests that tourism is a strategic pillar industry for China’s national economy and that promoting the integrated development of tourism and urbanisation is necessary.

However, persistent haze weather has occurred in many cities of China, attracting extensive public attention. The 2018 Communique on the State of China’s Environment shows that among 338 cities in China, 33.7% of cities meet the standard in ambient air quality and 66.3% of cities exceed the standard. The days in which PM2.5 (fine particles) is the primary pollutant account for 60.0% of the days with severe and above pollution. Owing to the vigorous reporting of social media and rapid dissemination of network information, the harm of haze weather, especially PM2.5, and its negative effects on public health [2,3], traffic safety [4,5] and city image [6,7] are widespread. At present, China’s worst-hit regions with large areas of haze are most centred on large- and medium-sized cities with developed social economies, and the tourism industry in these cities plays an important supporting role in social and economic development, considerably affecting the overall development of China’s tourism industry [8,9]. Therefore, studying the effect of haze weather on the efficiency of urban tourism development is of considerable practical significance. This paper aims to explore the effect of haze constraints characterised by PM2.5 emissions concentration on the temporal and spatial evolution of the efficiency of China’s urban tourism development and analyse the performance of urban tourism development. These goals are of major importance to deeply realise the quality of urban tourism development and promote the coordinated development of urban tourism and environment.

On this basis, this paper intends to evaluate the efficiency of urbanisation development of 58 Chinese cities in 2001–2016 by adopting the EBM super-efficiency model considering undesirable output and measure the total factor productivity by means of the Global Malmquist–Luenberger (GML) index [10] to provide the basis for the government to improve the efficiency of urban tourism development and formulate corresponding policies on urban tourism development.

## 2 Literature review

Studies on urban tourism development mainly focus on the connotation of urban tourism and the relationship between tourism and urbanisation. Urban tourism emerged after World War II. To meet the expanding needs of society for the functions of pleasure, vacation and retirement, urban tourism with high tourism resource endowment gradually increased and become a special form of urban renewal and evolution in places such as Florida, Arizona, California and Las Vegas in the United States. Mullins proposed the concept of tourism urbanisation in his study on Cold Coast and Sunshine Coast in Australia and firstly described the relationship between tourism and urbanisation theoretically [11]. The existing studies mainly elaborate on the connotation of tourism urbanisation from the perspective of Sociology of Consumption and holds that the rise of urban tourism is closely related with the social background. Tourism urbanisation originated from high income and mass consumption in the Fordism period in the early 20th century and matured in the 1970s, that is, the rise of mass hedonic consumption in the post-Fordism period. It is a form of postmodern cities [12]. Mullins believes that the formation mechanism of tourism urbanisation is mainly manifested as follows: the tourism industry can cause the expansion of urban population, the adjustment of urban industry structure and the changes in urban class structure. In particular, the rise of the modern service industry and the prominence of the petty bourgeoisie have become the main signs that tourism influences urbanisation [13]. Until the 1980s, with the advent of the post-industrial era, urban tourism activities began to be active. However, for example, some traditional industrial cities, such as Bradford in the UK, were transforming into tourism centres. Almost every city was boasting that it had at least one place with the most attractive tourism resources [14]. At the same time, urban tourism began to enter the field of vision of urbanology and tourism, and it was regarded as a ‘unique phenomenon and research field’ [15].

The urbanicity of the tourism industry and the modernity of cities provide superior conditions for the co-development of tourism and cities. In terms of the former, the coexistence of convenient transportation networks, shared service facilities and diversified product forms in the urban physical environment has laid the basis for the embedding of tourism functions; in terms of the latter, ‘future, risk and culture’ interweave in the urban physical environment, creating deepening conditions for strengthening ‘tourism functions’ [16,17]. Not only does a positive interaction occur between tourism and urbanisation, but also a potential negative influence. For example, the construction of travel reception ahead of the expansion of demand scale will lead to excessive urbanisation, and rapid urbanisation may also cause the deterioration of environmental quality and the destruction of ecological landscape [18–20], thereby negatively affecting the development of tourism [21–23].

Haze weather is regarded as an important manifestation of climate disaster [24,25]. Haze will directly influence tourists’ perception of comfort and health. Air pollution can cause asthma, bronchitis, emphysema pulmonum and other respiratory diseases [26,27] and significantly promote the incidence rate of respiratory diseases [28]. If severe haze weather occurs in the tourist destination, people’ worries about the health risk from travel will be increased, certainly considerably harming tourists’ choice of the destination and their revisit intention [29,30]. Furthermore, haze weather has a serious effect on people’s travel safety [31]. In haze weather, the drivers’ visual distance is reduced, and controlling vehicles is more difficult. Thus, misjudging the distance between vehicles is easy, thereby increasing traffic accident rates [32,33]. Haze brings major safety risks to travel. Haze will inevitably affect people’s perception of travel risk and will inevitably make haze weather become one of the important factors affecting tourists’ choice of the destination [34]. The image of tourist destination is multi-dimensional [35,36] and changing [37,38]. Climate condition is not only an attractive factor for tourist destinations but also a risk factor [6]. Climate change and air pollution have become major detriments to the tourism industry, considerably affecting the image of tourist destinations [39,40]. With the public’s wide attention to haze weather, once words related to haze become the hot descriptors for the image of tourist destinations, it will be seriously negatively affected. However, the image of a tourist destination has an important effect on the travel decision of potential tourists. The above analysis shows that climate, closely related to tourism, has become a decisive factor in the selection of tourist destination and vacation type. Climate has also indirectly affected the development of urban tourism.

Existing studies on the development of tourism efficiency mainly focus on the efficiency of tourism development factors. For example, in studies on the efficiency of tourism hotels, scholars aim to study the business position of tourism service market by using DEA from the evaluation on aspects such as hotel management performance and customer satisfaction assessment [41–43]. Then, more scholars focused on the influencing factors on the efficiency of tourism hotels [44,45]. The efficiency of travel agencies is also one of the important fields in the development of tourism efficiency. The results of most studies on the business efficiency of travel agencies show that the business efficiency of travel agencies is not high [46–48]. In addition, studies have been made on the efficiency of tourism transport and the operating efficiency of host cities [49,50]. In general, studies on the efficiency of tourism development have involved many fields, including hotels, travel agencies, tourism transport and tourism destinations. No systematic analysis and theoretical explanation have been conducted on the source of efficiency and generation mechanism, especially no the study on city-a comprehensive tourism destination, in these studies on the efficiency of tourism. Thus, taking the city, a complex integrated body, as a production unit, the efficiency of urban tourism is evaluated in this paper, and the characteristics of efficiency and its influencing factors are analysed. The existence of resource endowment enables the sustainable development of the tourism industry in cities. Furthermore, tourism, as a driving force, can promote the urbanisation process. However, haze weather in cities has many negative effects on the tourism industry; thus, measuring the efficiency of urban tourism development is of considerable significance.

Data envelopment analysis (DEA) is mainly adopted for existing studies on efficiency measurement methods and models. Charnes first proposed the DEA model [51]. This model has been widely used in research on environment and energy efficiency [52,53]. DEA can be divided into two categories. The first category is a DEA model that does not consider undesirable output. The evaluation model mainly includes the DEA and improved DEA models. The second category is the super-efficiency model, which is an SBM model considering undesirable output, that is, one or more pollutants are added to the first model as the undesirable output [54–56]. However, these methods cannot be used to reflect the actual production process, possibly leading to deviation of the results [57]. Later, Tone expanded the model [58] and added the relaxation of undesirable output into the objective function to modify the constraint conditions of undesirable output [59]. Combined with the development of research methods, some defects occur in the radial DEA and non-radial SBM models. Thus, in 2010, Tone proposed the EBM model, which is a hybrid model containing radial and SBM distance functions [60].

## 3 Materials and methods

### 3.1 Study area

**Fig 1:**
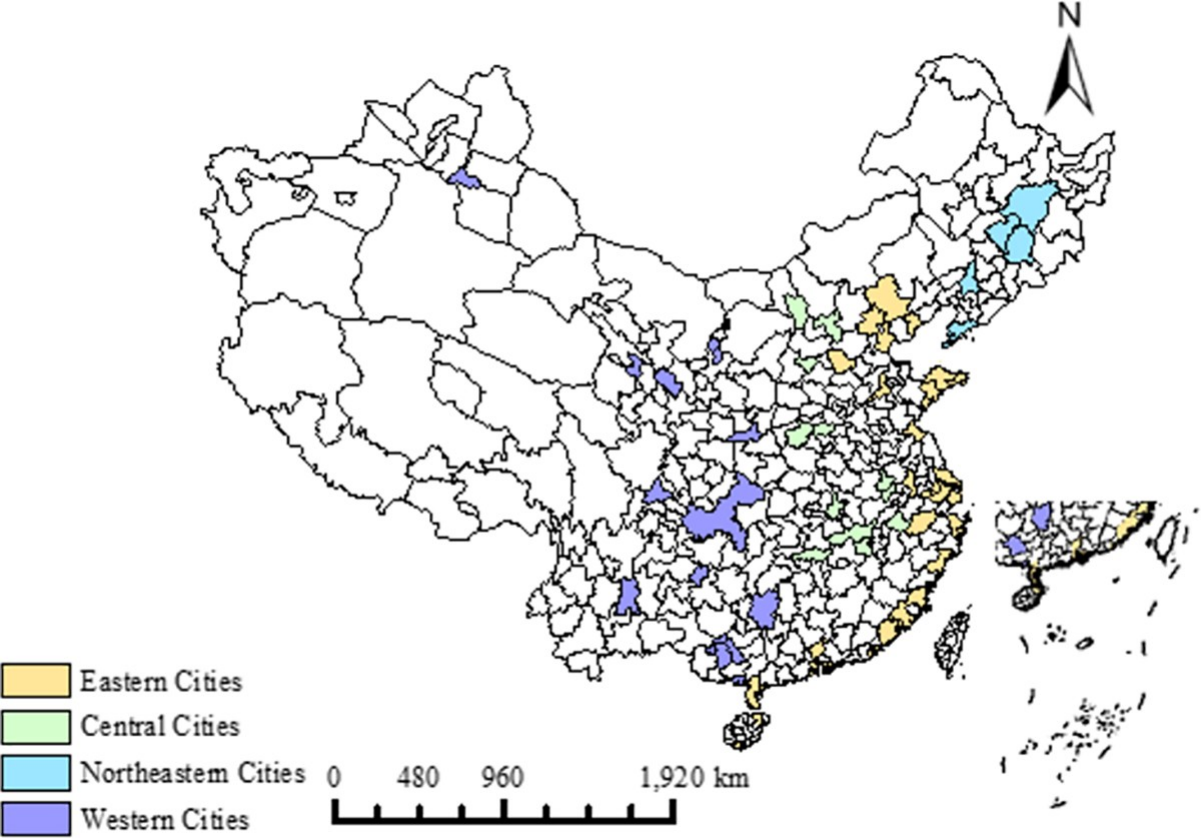
58 studied cities in China.

The China Tourism Statistical Yearbook provides long-term statistics on the level of tourism development in 60 cities, which are either provincial, municipal and autonomous regional capitals or famous scenic tourist cities, so they are called ‘major tourist cities ‘. In 2016,70.95 million tourists arrived in the 60 cities, accounting for 66.18 percent of inbound tourists in China. In terms of status and income, these cities represent the highest level of urban tourism development in China, which can reflect the real level of urban tourism development in China. The data are collected from the China Tourism Statistical Yearbook. Due the severely missing of indicator data, Yanbian and Lhasa are not included in this study. A total number of 58 main tourist cities are selected in this paper. These 58 cities are Beijing, Tianjin, Shijiazhuang, Qinhuangdao, Chengde, Taiyuan, Datong, Hohhot, Shenyang, Dalian, Changchun, Jilin, Harbin, Shanghai, Nanjing, Wuxi, Suzhou, Nantong, Lianyungang, Hangzhou, Ningbo, Wenzhou, Hefei, Huangshan, Fuzhou, Xiamen, Quanzhou, Zhangzhou, Nanchang, Jiujiang, Jinan, Qingdao, Yantai, Weihai, Zhengzhou, Luoyang, Wuhan, Changsha, Guangzhou, Shenzhen, Zhuhai, Shantou, Zhanjiang, Zhongshan, Nanning, Guilin, Beihai, Haikou, Sanya, Chongqing, Chengdu, Guiyang, Kunming, Xi’an, Lanzhou, Xining, Yinchuan and Urumqi. On the basis of the social and economic development level, the Chinese government divides the Chinese mainland (excluding Hong Kong, Taiwan and Macao) into four regions, namely, the northeastern, eastern, central and western regions. Fig 1 shows the regional distribution of 58 cities in China (*Figs 1 and 3 in the paper are due to the free maps provided by the National Geomatics Center of China at the* *http*:*//kmap*.*ckcest*.*cn/illustration/index*, *in which there is no copyright conflict*. *National Bureau of Statistics of China*. *Territorial division of eastern*, *western*, *central and northeastern regions*. *2011-06-03*. *China’s economic regions are divided into four regions*: *the East*, *the Central*, *the West and the Northeast*. *The eastern region includes Beijing*, *Tianjin*, *Hebei*, *Shanghai*, *Jiangshu*, *Zhejiang*, *Fujian*, *Shandong*, *Guangdong and Hainan; the central region includes Shanxi*, *Anhui*, *Jiangxi*, *Henan*, *Hubei and Hunan; the western region includes Inner Mongolia*, *Guangxi*, *Chongqing*, *Sichuan*, *Guizhou*, *Yunnan*, *Tibet*, *Shaanxi*, *Gansu*, *Qinghai*, *Ningxia and Xinjiang; the northeastern region includes Liaoning*, *Jilin and Heilongjiang*.). The common features of these cities is that all of them have superior human or natural resource endowment.

### 3.2 Data source and data pre-processing

#### 3.2.1 Urban tourism development-related data

In 2001, the Chinese government put the tourism industry on the agenda as an important industry to drive the new growth point of the national economy (*Central People’s Government of the People’s Republic of China*, *Notice of the State Council on Further Accelerating the Development of Tourism*, *Guofa No*.*〔2001〕9*. *http*:*//www*.*gov*.*cn/gongbao/content/2001/content_60814*.*htm*). This development indicates that China’s tourism has begun to become an important force to drive the national economy and social development. At the same time, restricted by the release date of research data, the research period of this paper was from 2001 to 2016. The data related to urban tourism development mainly came from the China Urban Statistical Yearbook and China Tourism Statistical Yearbook in the corresponding years and the National Economy and Social Development Statistical Bulletin in the corresponding cities.

#### 3.2.2 Urban haze contaminated data

Therefore, taking 58 tourism cities in China from 2001 to 2016 as the research samples, the PM2.5 concentration monitored by satellite was used to characterise haze contamination degree in this paper [61] (*Haze pollution is represented by the average PM2*.*5 concentration*, *data from raster data in PM2*.*5 concentration monitored with the global satellite released by Social and Economic Data and Application Center of Columbia University, https://beta.sedac.ciesin.columbia.edu/data/set/sdei-annual-pm2-5-concentrations-countries-urban-areas-v1-1998-2016*), and a systematic investigation was conducted on the efficiency of tourism development of tourism cities under the constraints of haze.

#### 3.2.3 Selection of measure indicators for the efficiency of urban tourism development and system construction

The efficiency of urban tourism development is one of the most important dimensions characterising its quality [62]. This dimension is mainly used to measure the use efficiency of various factors in the process of urban tourism promotion. The specific method in existing studies is to divide urban tourism into two systems, namely, input and output. Then, based on input and output, the efficiency of urban tourism development is calculated [63,64].

*3*.*2*.*3*.*1 Input indicators*. In the meaning of economics, the most basic production input factors include land, labour force and capital [65,66]. However, urban tourism production is not constrained by land area. Therefore, land cannot be used as an input variable of urban tourism development. However, the two factors, namely, labour force and capital, have important effects on the improvement of urban tourism satisfaction. Labour force is achieved through soft elements such as the service attitude and level of service personnel. The number of people directly employed in the tourism industry is an ideal indicator. However, affected by the comprehensive characteristics of tourism industry, the number is not officially counted in the relevant yearbooks. Finally, the number of employees in the tertiary industry, a macro and general indicator (this indicator has strong data availability and almost covers all the directly and indirectly employed people related to tourism industry, fully reflecting the comprehensive characteristics of tourism industry. The shortcoming is that it enlarges the input scale of actual factors) is adopted in this paper. Capital is achieved through hard elements, such as project construction, infrastructure improvement and tourism environment construction. Considering that China’s urbanisation development is not possible without the government’s financial capital investment [67,68], urban fixed-asset investment was used for characterisation in this paper. Furthermore, we analysed from the perspective of the improvement of urban tourism environment and investment directly used to improve tourists’ experience. Only the part for urban infrastructure and reconstruction has a strong causal relationship with the development of tourism. However, with the expansion of the connotations of tourism, the investment in urban real estate development and other investment in fixed assets represented by tourism real estate played an important role in the improvement of the overall attractiveness of urban tourism. Therefore, the development of urban tourism was enlarged to the industrial level in this paper, ignoring the differences in the use of urban fixed asset investment in different types of cities and the differences in the proportion of urban fixed asset investment directly used in tourism investment to the overall investment. At the same time, the foreign capital actually used in the practice is higher and the attraction of urban business tourism is stronger. Foreign direct investments reflect the scale of foreign exchanges and the intensity of foreign economic ties of a city. Furthermore, the amount of foreign capital introduced and used reflects the attraction of potential and comprehensive economic strength to foreign investors. The practice indicates that the cities with high foreign capital practically used often become important commercial centre cities and the locations of important multinational enterprises, where holding various types of foreign exchanges and various business affairs, exhibitions, conferences, festivals and entertainment activities, is easy. On the basis of these activities, the economic vitality of the city and the attractiveness of urban tourism are further improved [69].

*3*.*2*.*3*.*2 Output indicators*. The direct output of urban tourism should include the part meeting all needs and services of tourists in the process of travel [70,71]. However, tourism income and person-time of tourist reception are selected as the production output of tourism service in most of the literature on tourism efficiency [72–74], which are used to characterise the economic and scale levels of urban tourism development [75]. Total tourism income refers to the sum of domestic revenue and inbound revenue of each city at the end of the year. Total person-time of tourists refers to the sum of the person-time of domestic tourists and the person-time of inbound tourists of each city at the end of the year. The above paragraphs have also shown that the development of urban tourism has a profound relationship with haze [76]. We attempt to measure the efficiency of urbanisation development of 58 tourism cities in 2001–2016 under the constraint of haze pollution in this paper.

Therefore, the selection of measurable indicators for the efficiency of urban tourism development and system construction is shown in Table 1.

**Table 1:** Measure indicator system for the efficiency of urbanisation.

Types of indicators	Variables	Unit
**Input indicators**	Urban fixed-asset investment	10,000 CNY
	Employees in the tertiary industry	10,000 person
	Foreign direct investment	10,000 USD
**Output indicators**	Total urban tourism revenue	100 million CNY
	Total urban tourism population	10,000 person
**Undesirable output**	PM2.5 concentration	μg/m^3^

### 3.3 Methods

#### 3.3.1 Super-efficiency EBM model

DEA is a method for assessing the efficiency that can deal with problems on multi input–multi output simultaneously in the same framework. Given that the formal constraints of the specific functions in the stochastic frontier analysis are relaxed, it has a strong ability to explain the efficiency [52,53,66,77]. The traditional DEA model can be divided into two categories: the first comprises the radial models, namely, the CCR [51] and BCC models [78] proposed by Charnes, Cooper and other scholars; the second is the non-radial model proposed by Tone (2001). Namely, the SBM model [79]. With the traditional radial DEA model, all inputs and outputs must be reduced or expanded in the same proportion; thus, calculating slack variables is impossible. With the SBM model based on non-radial measurement, the non-radial slack variables are considered, but information on the proportion of the actual input–output value to the target value is lost. Against the defects of the traditional DEA model, Tone and Tsutsui proposed a hybrid model including radial and SBM distance functions, namely, the EBM model [60]. The advantage of the EBM model is that it can not only calculate the improvement ratio between the target and actual values but also find out the gap between the target and actual values through solving the non-radial value of each input and output. Therefore, it can measure the efficiency of the decision-making unit (DMU) accurately [80].

The EBM model can be divided into three types, namely, input-, output- and non-oriented. The input-oriented EBM model refers to the efficiency measurement being conducted based on the decrease of each input in the same proportion under the given output. The model emphasises that the output is unchanged, and the efficiency is maximised through changing the input indicators. The output-oriented EBM model refers to the efficiency measurement being conducted based on the increase of each output in the same proportion under the given input. The model emphasises that the input is unchanged, and the efficiency is maximised by changing the output indicators. The non-oriented EBM model aims to measure inefficiency from two aspects, namely, input and output. These three EBM models can be used to measure the relative efficiency of each DMU [81]. In this paper, the non-oriented EBM model that considers undesirable output is selected to evaluate the efficiency of China’s urbanisation development. The programming formula is as follows:

$$min\frac{\theta -\varepsilon_{x}(1/\sum_{i=1}^{m}\omega_{i}^{-})\sum_{i=1}^{m}\omega_{i}^{-}s_{i}^{-}/x_{ik}}{\varphi +\frac{\varepsilon_{y}(\frac{1}{\sum_{r=1}^{q}w_{r}^{g}})\sum_{r=1}^{q}\omega_{r}^{g}s_{r}^{g}}{y_{rk}}+\varepsilon_{z}(1/\sum_{i=1}^{p}\omega_{i}^{b})\sum_{t=1}^{p}\omega_{t}^{b}s_{t}^{b}/z_{tk}}$$ (1)

$$s.t.\left\{ \begin{array}{c} X\gamma +s_{i}^{-}=\theta x_{k}\\ Y^{g}\gamma -s_{r}^{g}=\varphi y_{k}\\ Z^{b}\gamma +s_{t}^{b}=\varphi z_{k}\\ \gamma ,s_{i}^{-},s_{r}^{g},s_{t}^{b}\geq 0 \end{array}\right.$$

In this programming formula, m, q and p refer to the amount of input, desirable output and undesirable output, respectively. *X*, *Y*^*g*^ and *Z*^*b*^ refer to input vector, desirable output vector and undesirable output vector, respectively. *x*_*ik*_, *y*_*rk*_ and *z*_*tk*_ refer to input, desirable output and undesirable output of decision-making unit *k*, respectively. $s_{i}^{-},s_{r}^{g}$ and $s_{t}^{b}$ refer to slack of input, desirable output and undesirable output, respectively. $\omega_{i}^{-},\omega^{g}$ and ω^b^ refer to the relative importance of each input indicator, desirable output and undesirable output respectively. *θ* refers to radial efficiency value. *ε*_*x*_, *ε*_*y*_ and *ε*_*z*_ refer to core parameters corresponded by radial *θ* and non-radial slack $s_{i}^{-},s_{r}^{g}$ and $s_{t}^{b}.$ *φ* refers to the planning parameter of the radial part of the output indicator. *γ* refers to the linear combination coefficient of the decision-making unit. ε is valued in the range of [0,1], and it is a key parameter, representing the importance of the non-radial part in the measurement of efficiency. If ε = 1, the EBM model is equivalent to the SBM model. If ε = 0, the EBM model is equivalent to the radial DEA model.

In the analysis result of the DEA model, many decision-making units (DMUs) are often evaluated as effective. In particular, when the input and output indicators in consideration are numerous, the number of effective DMUs will increase accordingly. In the DEA model, the maximum efficiency value obtained is 1, and the effective DMU efficiency value is the same. Further distinguishing the efficiency value of DMU is difficult. To solve this problem, Andersen and Petersen [82] proposed a method, called the Super Efficiency Model, to further distinguish the effective DMUs. For easy differentiation, the traditional DEA model is called the Standard Efficiency Model. The unique different point of the former from the latter is that the constraint condition j≠k is added. The programming formula of the Super Efficiency Model is as follows:

$$\begin{array}{l} min\theta \\ s.t.\left\{ \begin{array}{c} \sum_{\begin{array}{c} j=1\\ j\neq k \end{array}}^{n}\gamma_{j}x_{ij}\leq \theta x_{ik}\\ \sum_{\begin{array}{c} j=1\\ j\neq k \end{array}}^{n}\gamma_{j}y_{rj}\geq y_{rk}\\ \gamma \geq 0\\ i=1,2,\ldots ,m;r=1,2,\ldots ,q;j=1,2,\ldots ,n(j\neq k) \end{array}\right. \end{array}$$ (2)

Here, the meaning of each variable is the same as that of the previous variable. The core of the Super Efficiency Model is that the DMU evaluated is removed from the reference set. Therefore, the efficiency value of the DMU evaluated is obtained by referring to the frontier formed by the remaining DMUs. The effective DMU value is generally more than 1 to distinguish the effective DMUs.

The super-efficiency EBM model is based on the cross-sectional data, so it can only measure the efficiency value of each DMU at one time point, introducing difficulties in comparing the calculation results of data at different time points. Therefore, we attempt to combine it with the GML index analysis to conduct a dynamic analysis of time in this paper.

#### 3.3.2 Construction of directional distance function

Chung et al. applied directional distance function containing undesirable output to the Malmquist model and collectively referred to the Malmquist index as the Malmquist–Luenberger productivity index [83]. This index can effectively solve the problems on the efficiency evaluation of undesirable output. The programming formula of this index is as follows:

$$\underset{D^{t}}{\to }(x^{t},y^{t},b^{t};g)=sup\;\{\beta :(y^{t},b^{t})+\beta g\in P^{t}(x^{t})\}$$ (3)

In the formula, x^t^ represents land, labour force, capital and other input vectors. y^t^ and g_y_ represent the vectors of desirable output at Phase t. b^t^ and g_b_ represent the vectors of undesirable output at Phase t. g = (g_y_, g_b_) refers to a directional vector. β is a directional distance function of maximising desirable output and minimising undesirable output at Phase t [84]. Assuming that nDMUs (cities) are used, each DMU has i inputs x = $(x_{1},x_{2},\ldots ,x_{i})\in R_{i}^{+}$. Then, u desirable outputs y = $(y_{1},y_{2},\ldots ,y_{u})\in R_{u}^{+}$ and z undesirable outputs b = $(b_{1},b_{2},\ldots ,b_{u})\in R_{z}^{+}$ are obtained. P^t^(x) refers to production possibility set in t = 1,…,at T times:

$$P^{t}(x)=\{(y^{t},b^{t}):x^{t}\overset{produce}{\Rightarrow }(y^{t},b^{t})\},\;x\in R_{i}^{+}$$ (4)

ML productivity can be generally divided into two parts: ${MLTECH}_{t}^{t+1}$ representing the technical progress index and ${MLEFFCH}_{t}^{t+1}$ representing the efficiency change index. In other words, ${ML}_{t}^{t+1}={MLTECH}_{t}^{t+1}\times {MLEFFCH}_{t}^{t+1}$. In the formula, ML, MLTECH, MLEFFCH>1 or (<1) represent the growth (drop), technical progress (retrogress) and efficiency improvement (deterioration) of total factor productivity, respectively.

#### 3.3.3 GML index model

TFP (total factor productivity) calculated by adopting the ML index in the form of average geometry does not have the characteristics of cyclic multiplication. Therefore, we can only analyse the short-term change of adjacent production efficiency, but we cannot observe the long-term growth of production efficiency. In addition, the directional distance function easily incurs problems without feasible solutions [85]. Therefore, Oh constructed the GML index model, which takes the sum of common reference sets at different phases as a reference set [86]. The common reference set at all phases is $S^{g}=S^{1}\cup S^{2}\cup S^{3}{\cup S}^{4}\cup \ldots {\cup S}^{p}=\{(x_{j}^{1},y_{j}^{1})\}\cup \{\left(x_{j}^{2},y_{j}^{2}\right)\}\cup \ldots \{(x_{j}^{t},y_{j}^{t})\}$. The GML index method can be used to avoid the possibility of technical retrogression. In addition, because the DMU evaluated is certainly included in the global reference set, the GML index does not have a problem without a feasible solution. Furthermore, the GML index is characterised by being transitive and able to be multiplied. In this paper, based on the idea of Oh [86], we combine the GML index with the EBM super-efficiency model to construct the programming formula of the GML index from Phase t to Phase t+1 as follows:

$${GML}_{t}^{t+1}(x^{t},y^{t}{,b}^{t},x^{t+1}{,y}^{t+1},b^{t+1})=\frac{1+D^{G}(x^{t},y^{t}{,b}^{t})}{1+{D^{G}(x}^{t+1}{,y}^{t+1},b^{t+1})}$$ (5)

We can further decompose the GML index into efficiency change (GEFC) and technical change (GETC). The programming formula is as follows:

$$\begin{array}{l} \;{GML}_{t}^{t+1}(x^{t},y^{t}{,b}^{t},x^{t+1}{,y}^{t+1},b^{t+1})\\ =\frac{1+D^{t}(x^{t},y^{t}{,b}^{t})}{1+D^{t+1}(x^{t+1}{,y}^{t+1},b^{t+1})}\times \left[\frac{1+D^{G}(x^{t},y^{t}{,b}^{t})}{1+D^{t}(x^{t}{,y}^{t},b^{t})}\times \frac{1+D^{t+1}(x^{t+1},y^{t+1}{,b}^{t+1})}{1+D^{G}(x^{t+1}{,y}^{t+1},b^{t+1})}\right] \end{array}$$ (6)

The decomposition method of Zofio can be regarded as an extension of the decomposition method of Fare R et al [87,88]. Based on the Zofio productivity decomposition method, GEFC is further decomposed into pure efficiency change (GPEC) and scale efficiency change (GSEC). Then, GTEC is further decomposed into pure technical change (GPTC) and technical scale change (GSTC), as follows:

$${GML}_{t}^{t+1}={GPEC}_{t}^{t+1}+{GSEC}_{t}^{t+1}+{GPTC}_{t}^{t+1}+{GSTC}_{t}^{t+1}$$ (7)

On the basis of different results, GPEC is used to reflect the level of environmental governance of urban development. GSEC refers to the scale economy of urban development. GPTC is used to distinguish the standards for technical progress in different regions. GSTC reflects the interaction between economic scale and technical progress. GML, GPEC, GSEC, GPTC and GSTC>1 or (<1) represent the growth or decline of total factor productivity of urbanisation development, the improvement or retrogression of environmental governance, the growth or decline of scale economy, the progress or retrogress of technology and the improvement or deterioration of the interaction relationship between economic scale and technical progress, respectively. We can analyse the efficiency of urban development by using this decomposition method from four dimensions in this paper. This study is more comprehensive and more concrete than previous studies.

## 4 Research and analysis

### 4.1 Analysis of the overall change characteristics of the efficiency of urban tourism development under the constraint of haze

The spatial and temporal differentiation characteristics of urban development efficiency under the constraint of undesirable output is observed according to the calculation result of the EBM model.

#### (1) Analysing from evolution over time

The efficiency of tourism development of 58 cities has been steadily improved in 2001–2016 but declined in 2002–2003. It’s shows a fluctuated upward trend. While the average PM2.5 emission reached the peak value of 71.2 in 2012, and it was relatively stable in other years.

**Fig 2:**
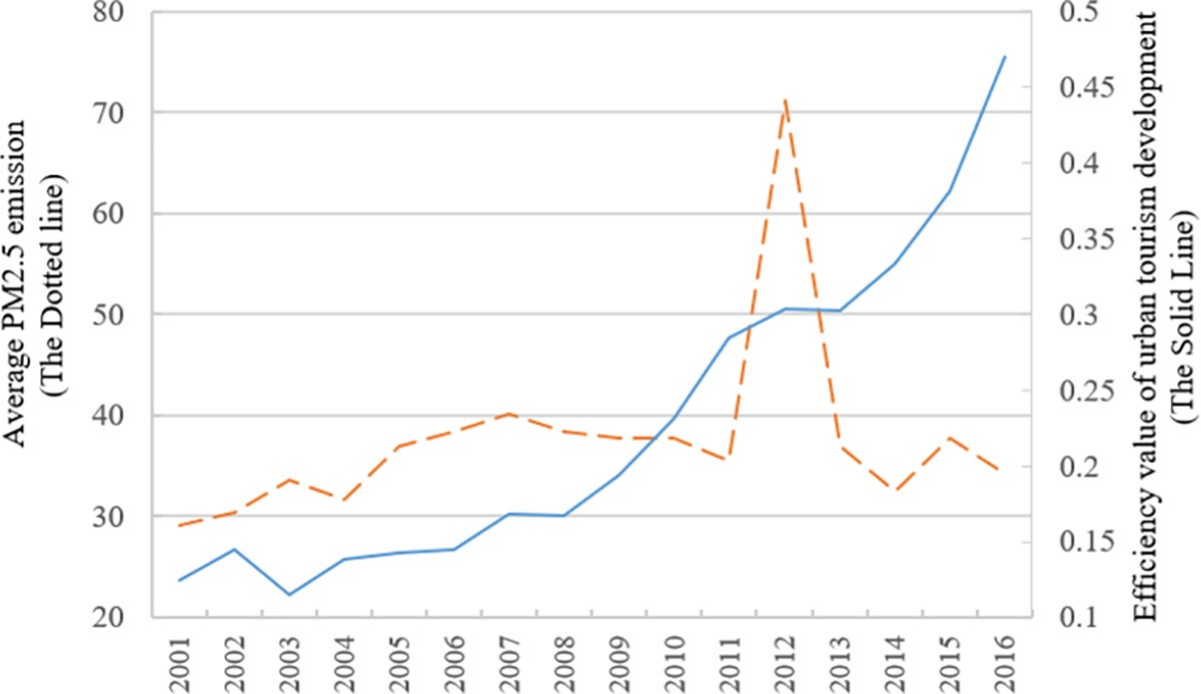
Variation of the efficiency of urban tourism development and PM2.5 emissions in 2001–2016.

Fig 2 shows curves of the efficiency of urban tourism development and PM2.5 emissions in 58 cities over time. The figure shows that the average efficiency of urban tourism development of 58 cities has increased 0.12 to 0.47 over 16 years. This finding indicates that the resource input by 58 cities have increasingly higher effects on tourism development, but the overall efficiency is not high. The findings also reveal that the efficiency of urban tourism development has not been improved rapidly with the rapid development of urbanisation under the constraint of haze.

#### (2) Analysing from the city level

**Fig 3:**
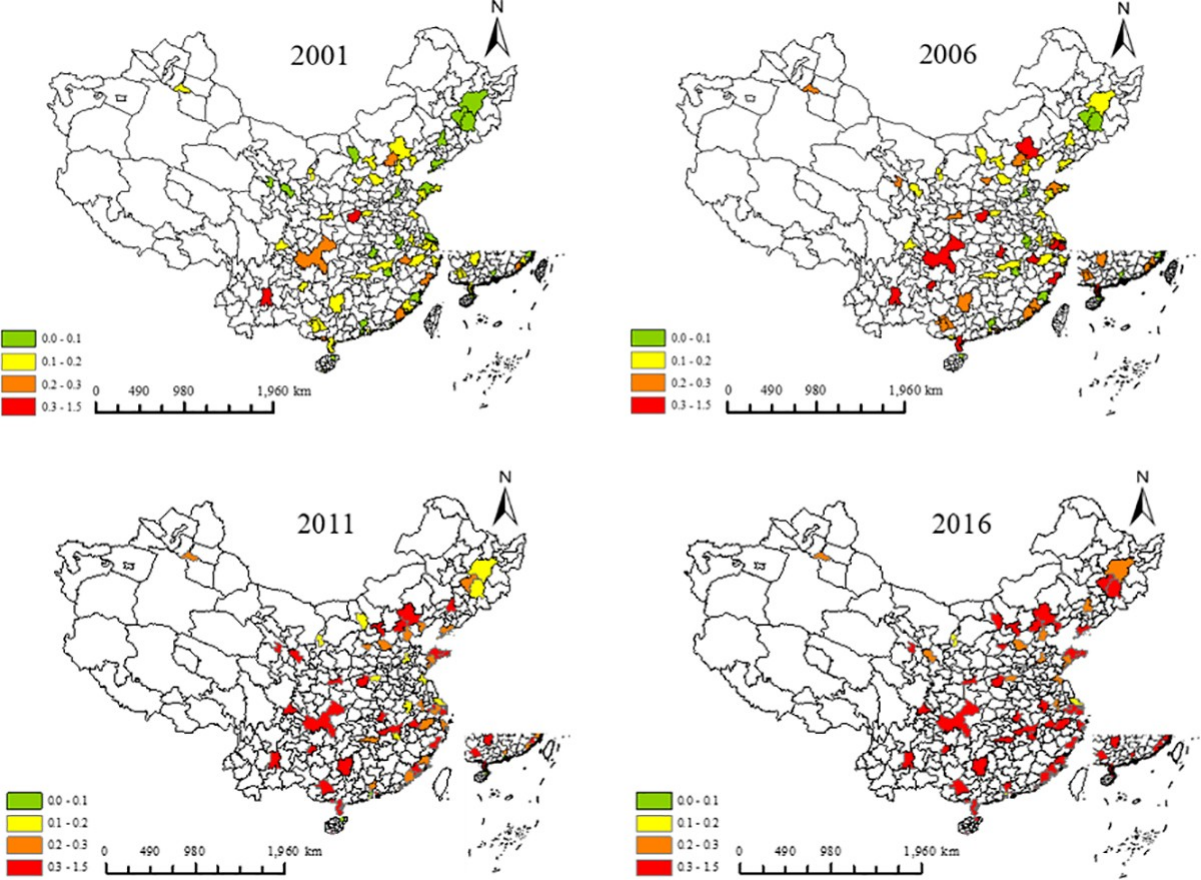
Urban tourism efficiency of 58 cities in 2001, 2006, 2011 and 2016.

In this part, the calculation results of the efficiency of urban tourism development are relatively low. Against the efficiency of urban tourism development, 0.1, 0.2, 0.3 and 1.5 are used as the threshold values for classification. The efficiency is valued in (0,0.1]), (0.1,0.2), (0.2,0.3) and (0.3,1.5), respectively, to conduct classification, as shown in (Fig 3).

The average efficiency of urban tourism development of 58 cities in 2001, 2006, 2011 and 2016 shows that the efficiency of tourism development of each city is improving. Under the constraint of haze, the efficiency of urban tourism development is not directly proportional to the development of the city. For example, Beijing, Shanghai and Tianjin are less efficient. However, in many cities in the western region with less haze weather and good ecological environment, the efficiency of urban tourism development is higher than that in the cities in the eastern, central and northeastern regions. Therefore, the input of all production factors should be strengthened in urban tourism development to improve the efficiency of the development. At the same time, paying attention to the governance of polluted weather is necessary to reduce the urban haze days and create a good environment for the development of urban tourism.

### 4.2 Analysis of the improvement of the efficiency of urban tourism development under the constraint of haze

The advantages of the EBM model is that it can measure the input redundancy, insufficient quantity of desirable output and the redundancy of undesirable output from the perspective of proportional improvement value and slack improvement value. The sum of these two values is the total redundancy value. The analysis on the redundancy (deficiency) of input and output indicators can reflect the cause for efficiency loss, helping to provide direction for improving the efficiency of urban tourism development. In this paper, the average value of redundancy (deficiency) of all indicators of 58 cities in 2001–2016 is divided by the average value of corresponding input (output) indicators. Then, the input redundancy rate and output deficiency (redundancy) rate of all indicators are obtained. The calculation results are shown in Tables 2–5. At the same time, the area in which cities are located is divided into the eastern, northeastern, central and western regions. The input-output redundancy rate is shown in Fig 2.

**Table 2:** Input–output optimisation result of tourism development in the main western cities.

Cities	Input redundancy rate (%)			Output deficiency rate (%)		
	I_1_	I_2_	I_3_	O_1_	O_2_	O_3_
Beihai	−64.66	−66.83	−62.74	77.06	**51.42** ^*^	**−79.80** ^*^
Chengdu	−63.93	−62.92	−61.45	136.65	33.45	−49.66
Guiyang	−51.91	−46.03	−35.15	116.95	35.84	−48.24
Guilin	−50.23	−50.48	−43.89	59.95	33.73	−49.74
Hohhot	−78.43	−71.12	−56.57	18.80	19.90	−66.85
Kunming	−59.49	−62.61	−52.55	204.73	26.74	−40.48
Lanzhou	−73.70	−67.49	−59.34	**524.93** ^*^	34.89	**−67.44** ^*^
Nanning	−45.17	−52.47	−44.92	150.21	34.92	−48.97
Urumqi	−68.89	−72.16	−63.86	111.67	**63.93** ^*^	−65.81
Xi’an	−79.14	−65.65	−73.03	**374.68** ^*^	48.91	−60.77
Xining	−60.82	−68.26	−61.05	232.91	50.51	−61.60
Yinchuan	−85.33	−84.49	−77.72	113.19	**79.46** ^*^	**−81.95** ^*^
Chongqing	−45.39	−63.01	−35.59	**253.99** ^*^	5.90	−28.34

Note: In Table 2, I_1_ refers to investment in fixed assets, I_2_ refers to employees in the tertiary industry, I_3_ refers to foreign direct investment, O_1_ refers to total tourism income, O_2_ refers to total tourism population and O_3_ refers to PM2.5 emission concentration. 1) The negative number in the table represents that this indicator is redundant, and the positive number represents that this indicator is insufficient. Redundancy (deficiency) rate refers to the absolute value of the corresponding value of each indicator. 2) The dark colour blocks in the table represent the indicator ranking first in the redundancy rate. 3) The figures with ‘*’ in the output indicators correspond to the cities in the top three of deficiency (redundancy) rate.

**Table 3:** Input–output optimisation result of tourism development in the main central cities.

Cities	Input redundancy rate (%)			Output deficiency redundancy rate (%)		
	I_1_	I_2_	I_3_	O_1_	O_2_	O_3_
Datong	−70.11	−62.24	−53.71	83.76	45.33	−59.74
Hefei	−86.88	−71.83	−79.12	119.26	63.54	**−76.53** ^*^
Huangshan	−19.50	−51.63	−21.61	30.81	10.56	−64.59
Jiujiang	−50.61	−55.33	−37.85	71.67	20.58	−60.68
Luoyang	−58.29	−34.12	−49.82	**234.56** ^*^	27.87	−33.34
Nanchang	−89.23	−77.36	−87.10	212.20	**65.41** ^*^	**−80.18** ^*^
Qinghuangdao	−69.20	−72.99	−75.47	96.13	**64.88** ^*^	**−72.75** ^*^
Shijiazhuang	−72.89	−70.56	−62.56	**243.76** ^*^	61.08	−67.63
Taiyuan	−73.79	−67.53	−63.69	**243.54** ^*^	58.27	−64.91
Wuhan	−84.51	−58.14	−78.60	232.26	40.33	−59.84
Changsha	−75.60	−57.45	−74.47	210.84	50.72	−60.12
zhengzhou	−82.73	−68.68	−76.62	185.14	**65.04** ^*^	−70.21
Chengde	−36.33	−−48.13	−36.07	36.15	13.41	−45.61

Note: In Table 3, I_1_ refers to investment in fixed assets, I_2_ refers to employees in the tertiary industry, I_3_ refers to foreign direct investment, O_1_ refers to total tourism income, O_2_ refers to total tourism population and O_3_ refers to PM2.5 emission concentration. 1) The negative number in the table represents that this indicator is redundant, and the positive number represents that this indicator is insufficient. Redundancy (deficiency) rate refers to the absolute value of the corresponding value of each indicator. 2) The dark colour blocks in the table represent the indicator ranking first in the redundancy rate. 3) The figures with ‘*’ in the output indicators correspond to the cities in the top three of deficiency (redundancy) rate.

**Table 4:** Input–output optimisation result of tourism development in the main eastern cities.

Cities	Input redundancy rate (%)			Output deficiency redundancy rate (%)		
	I_1_	I_2_	I_3_	O_1_	O_2_	O_3_
Beijing	−80.15	−80.49	−53.37	75.79	32.92	−36.29
Fuzhou	−87.20	−76.57	−73.74	**206.28** ^*^	59.96	−72.63
Guangzhou	−93.09	−82.15	−86.91	67.31	**110.85** ^*^	−67.70
Haikou	−87.32	−83.69	−86.55	**216.48** ^*^	**82.47** ^*^	−82.72
Hangzhou	−78.95	−60.51	−70.73	102.66	53.93	−60.60
Lianyungang	−73.81	−77.11	−79.43	73.20	71.63	**−84.77** ^*^
Nanjing	−89.86	−65.09	−81.22	128.00	62.70	−66.69
Nantong	−84.30	−79.81	−85.10	75.36	75.60	**−83.14** ^*^
Ningbo	−85.92	−59.32	−80.81	141.90	55.14	−62.40
Qingdao	−88.52	−69.22	−86.28	78.22	66.29	−69.89
Quanzhou	−59.84	−58.92	−58.38	134.90	47.99	−58.78
Sanya	−72.98	−75.72	−75.57	55.28	56.94	−70.02
Xiamen	−91.50	−58.06	−84.26	130.08	56.49	−62.55
Shantou	−82.68	−79.98	−79.21	77.36	**86.39** ^*^	−78.99
Shanghai	−73.12	−60.17	−67.00	73.59	46.01	−52.05
Shenzhen	−86.41	−60.20	−71.59	**270.42** ^*^	51.93	−53.72
Suzhou	−82.64	−51.80	−88.11	57.39	46.38	−53.19
Tianjin	−87.59	−65.02	−93.05	141.57	60.38	−65.17
Weihai	−67.74	−65.56	−84.83	61.55	61.14	−74.52
Wenzhou	−35.31	−44.27	−41.15	87.45	23.70	−44.84
Wuxi	−81.39	−54.44	−88.84	48.72	50.27	−58.30
Yantai	−82.13	−67.87	−71.06	170.47	61.31	−69.27
Zhanjiang	−63.39	−64.60	−63.59	126.44	40.12	−58.22
Zhangzhou	−65.19	−63.16	−58.14	119.13	54.29	−64.59
Zhongshan	−96.38	−79.66	−93.61	78.36	77.74	**−90.15** ^*^
Zhuhai	−88.37	−63.18	−89.99	126.79	62.68	−65.11
Jinan	−82.27	−76.69	−74.70	154.80	72.33	−77.30

Note: In Table 4, I_1_ refers to investment in fixed assets, I_2_ refers to employees in the tertiary industry, I_3_ refers to foreign direct investment, O_1_ refers to total tourism income, O_2_ refers to total tourism population and O_3_ refers to PM2.5 emission concentration. 1) The negative number in the table represents that this indicator is redundant, and the positive number represents that this indicator is insufficient. Redundancy (deficiency) rate refers to the absolute value of the corresponding value of each indicator. 2) The dark colour blocks in the table represent the indicator ranking first in the redundancy rate. 3) The figures with ‘*’ in the output indicators correspond to the cities in the top three of deficiency (redundancy) rate.

**Table 5:** Input–output optimisation result of tourism development in the main northeastern cities.

Cites	Input redundancy rate (%)			Output deficiency redundancy rate (%)		
	I_1_	I_2_	I_3_	O_1_	O_2_	O_3_
Dalian	−86.67	−65.26	−93.47	**187.29** ^*^	58.73	−66.26
Harbin	−76.27	−73.72	−73.98	**238.13** ^*^	**65.08** ^*^	**−67.83** ^*^
Jilin	−92.65	−87.45	−95.58	102.88	**64.10** ^*^	**−75.64** ^*^
Shenyang	−84.84	−65.64	−87.19	**280.14** ^*^	58.79	−62.49
Changchun	−80.12	−72.83	−81.25	150.02	**59.82** ^*^	**−69.58** ^*^

Note: In Table 5, I_1_ refers to investment in fixed assets, I_2_ refers to employees in the tertiary industry, I_3_ refers to foreign direct investment, O_1_ refers to total tourism income, O_2_ refers to total tourism population and O_3_ refers to PM2.5 emission concentration. 1) The negative number in the table represents that this indicator is redundant, and the positive number represents that this indicator is insufficient. Redundancy (deficiency) rate refers to the absolute value of the corresponding value of each indicator. 2) The dark colour blocks in the table represent the indicator ranking first in the redundancy rate. 3) The figures with ‘*’ in the output indicators correspond to the cities in the top three of deficiency (redundancy) rate.

From the perspective of input indicators, the overall redundancy rates of 58 cities in various input indicators are relatively high. The redundancy rates of input indicators of most cities are more than 50%. This finding that most of the resource inputs do not play an actual role in promoting urban tourism development, resulting in a waste of resources. Tables 2–5 clearly show that the redundancy of the investment in fixed assets (I_1_) in most cities is the primary factor affecting the efficiency loss of urban tourism development, followed by foreign direct investment (I_2_). The investment in fixed assets will considerably improve the construction of urban tourism infrastructure and its supporting facilities. However, the situation of extensive construction and disorderly expansion is also a problem in China’s urbanisation development, wasting resources. Foreign direct investment will further aggregate tourism development factors in different forms, such as the stream of people, circulation of materials, flow of funds and information flow. However, the tourism reception facilities and carrying capacity of cities will also bear great pressure, hindering the improvement of tourism development efficiency in turn. Therefore, we should rationally plan the resource input of these indicators in the future, optimise the resource input in urban tourism development, balance the resource input by combining the demand of urban development and attempt to avoid unnecessary waste of resources.

From the perspective of output indicators, major differences in output deficiency (redundancy) rate occur among cities. These differences are related to the resource endowment of different cities. First, the desirable output deficiency rate of total tourism income (O_1_) is relatively high, whereas the desirable output deficiency rate of total tourism population (O_2_) is relatively low. This finding indicates that the economic effect of urban tourism development is not good, and the attraction to tourists is also general, but it is better than total tourism income. The undesirable output PM2.5 emission concentration (O_3_) is generally high. A comparison of the desirable output deficiency rate and undesirable output redundancy rate among different cities shows that excessive PM2.5 emissions have a relatively major effect on the efficiency loss of urban tourism development. In addition, compared with the redundancy rate of input indicators, output deficiency (redundancy) rate is generally high. Therefore, we should focus on the improvement of various output indicators and the decrease in PM2.5 emission concentration in the improvement of urban tourism development efficiency to attract tourists and increase tourism income.

**Fig 4:**
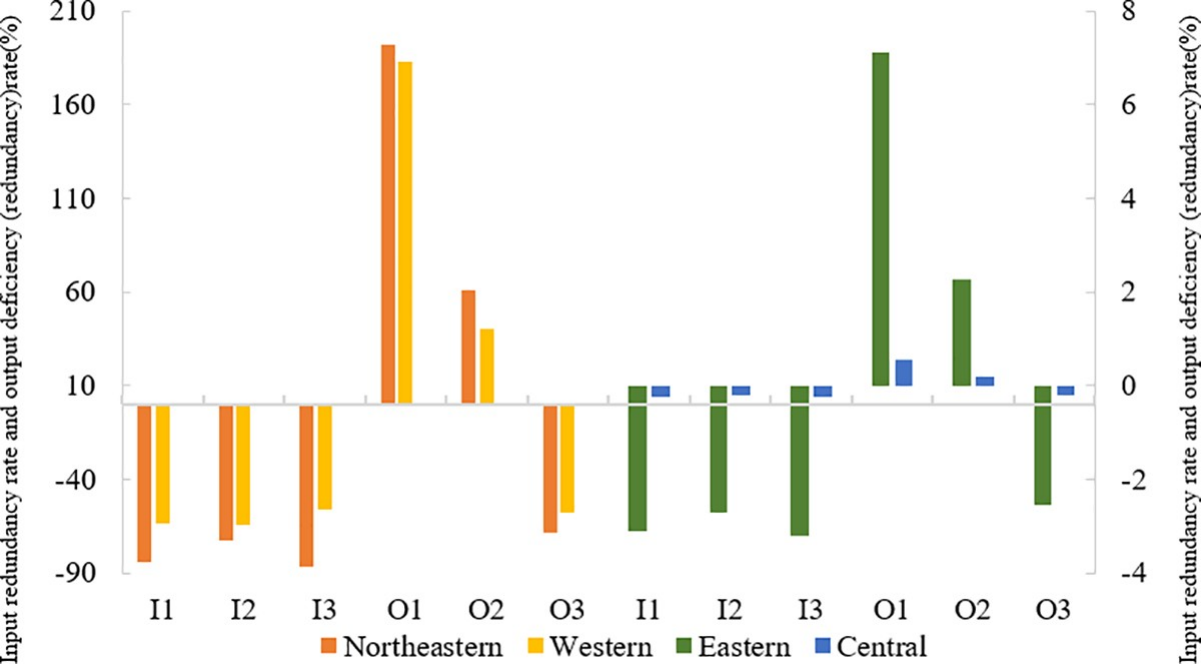
Input–output optimisation result of tourism development based on the regions where cities are located.

In this section, based on the regions in which China’s 58 cities are located (Fig 4), the characteristics of spatial differentiation of input redundancy rate and output deficiency (redundancy) rate of China’s cities in 2001–2016 are analysed as follows. On the whole, the input redundancy rate and output deficiency (redundancy) rate of cities in the northeastern and western regions are much higher than those in the central and eastern regions. On the one hand, the tourism development level of cities in the northeastern and western regions is lower than that in the central and eastern regions. On the other hand, the development of cities in the northeastern region depends on heavy industry, and the development of cities in the western region highly depends on the energy and resource input. These results also make PM2.5 emission concentration in these regions high and affect the efficiency of urban tourism development. At the same time, the redundancy rate of the two regions is not significantly different. Furthermore, input redundancy rate and output deficiency (redundancy) rate of tourism development in the eastern cities are far higher than those in the central regions, which is closely related to excessive input factors and low output level of urban tourism development in the eastern region. However, in the central region, input redundancy rate and output deficiency (redundancy) rate of urban tourism development are relatively balanced. Therefore, rationally allocating various input resources according to the characteristics of regional tourism development in the formulation of macro-regional tourism policies and paying attention to the effect of PM2.5 emission on the efficiency of tourism development are necessary.

### 4.3 Analysis of total factor productivity in urban tourism development under the constraint of haze

**Fig 5:**
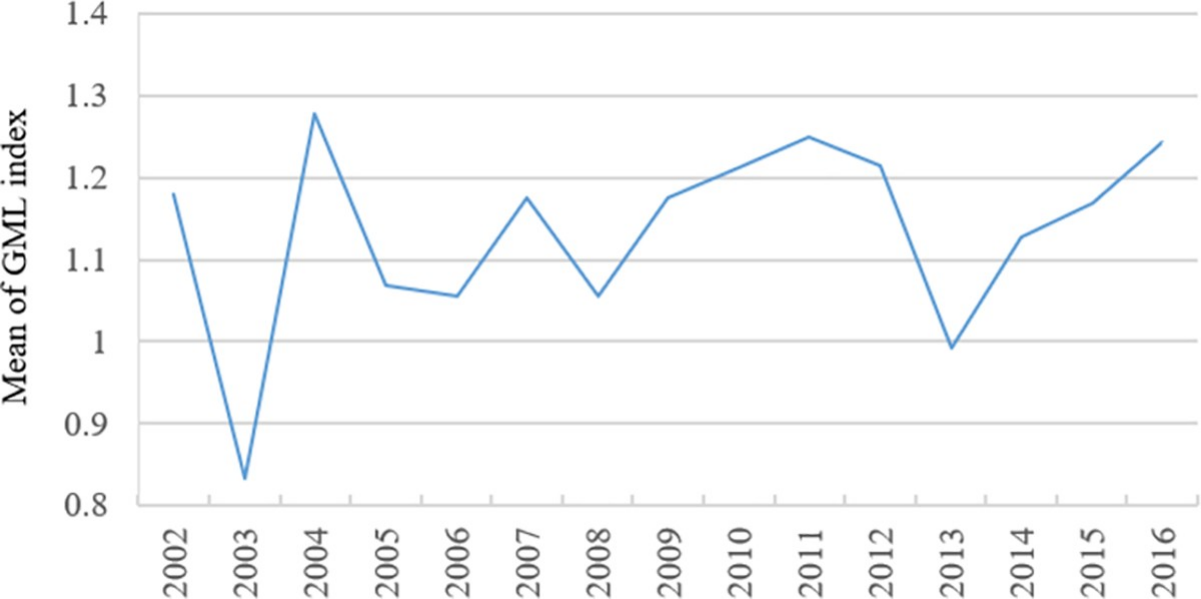
GML index change in tourism development of major cities in China in 2002–2016.

**Fig 6:**
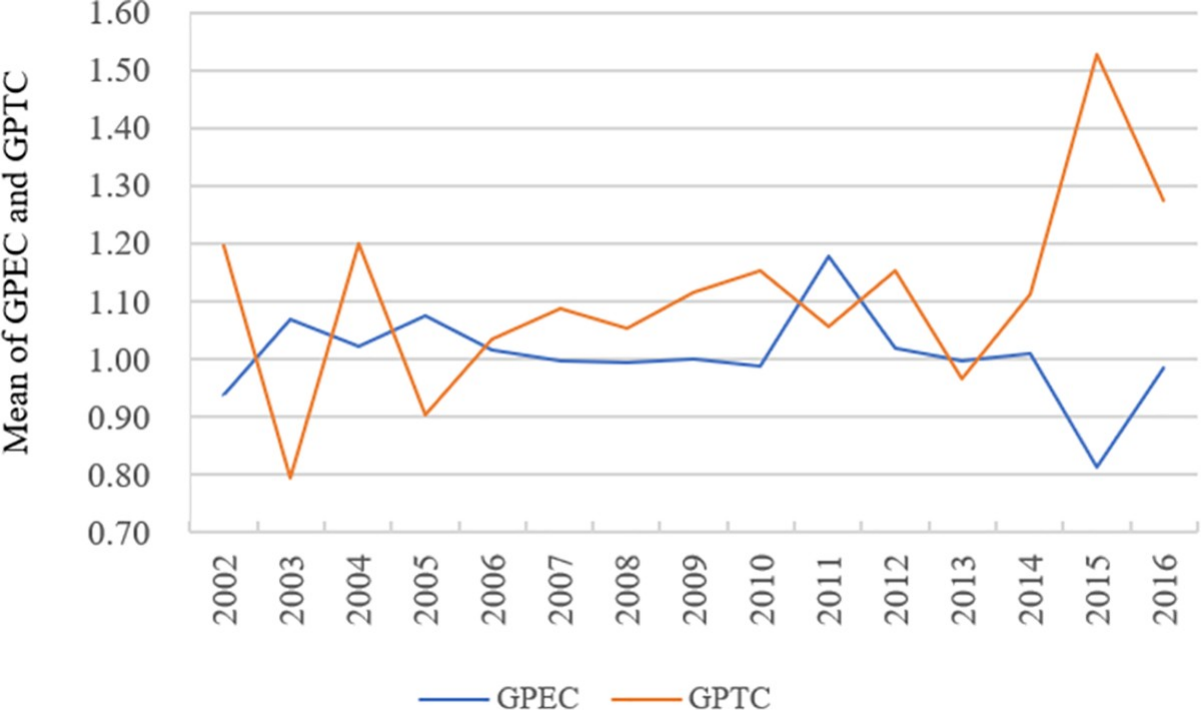
Changes in GPEC and GPTC in tourism development of major cities in China in 2002–2016.

**Fig 7:**
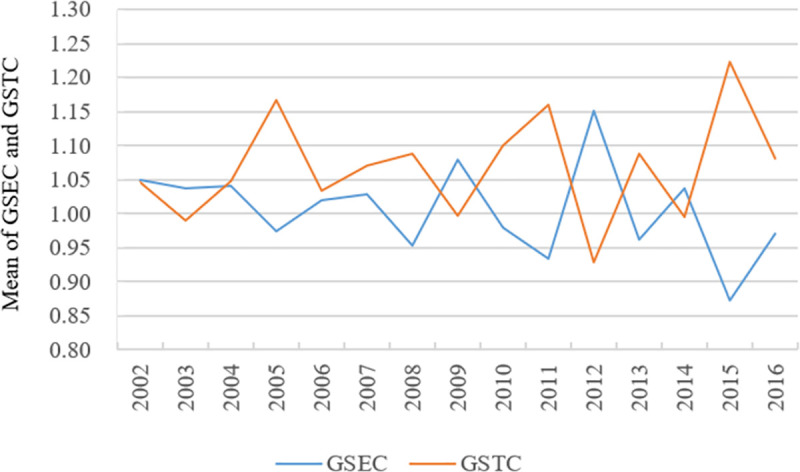
Changes in GSEC and GSTC of tourism development in China’s major cities in 2002–2016.

Total factor productivity (TFP), regarded as an important index to evaluate the economic growth rate, refers to the ‘efficiency of production activities in a certain period of time’, and it is a productivity index to measure the total output per unit of total input. Based on the GML index analysis method, we measure the total factor productivity of tourism development in 58 major cities in China in 2001–2016, in consideration of the undesirable output of PM2.5 in this paper. We decompose them into GPE, GSEC, GPTC and GSTC based on the Zofio productivity decomposition method to analyse the driving factors for total factor productivity change in tourism development. The calculation results are shown in Figs 5–7.

Fig 5 shows the geometric mean of GML index in tourism development of major cities in China in 2002–2016 (*The average value refers to the arithmetic mean unless otherwise stated. According to Oh [24] (2010), the geometric mean is better than the arithmetic mean in GML index analysis. In Fig 4, GML indexes of different years correspond to the geometric mean values of GML indexes of tourism development in 58 major cities in 14 stages, namely, 2002–2003, 2003–2004, 2004–2005, 2005–2006, 2006–2007, 2007–2008, 2008–2009, 2009–2010, 2010–2011, 2011–2012, 2012–2013, 2013–2014, 2014–2015 and 2015–2016*). The GML index value of tourism development of major cities in China shows a fluctuated upward trend in 2002–2016, under the constraint of PM2.5. However, on the whole, only the corresponding GML indexes in 2003 and 2013 are less than 1 (0.83 and 0.99, respectively), and the GML indexes in other stages are greater than 1. A comparison with Fig 2 shows that during the period from 2001 to 2016, the efficiency of tourism development in 2002–2003 and 2012–2013 is slightly decreased, which is closely related to the SARS outbreak in China in the spring of 2003 when China’s tourism was seriously hit, and the transition of China’s economic and social development to the ‘New Normal’ in 2013(*Xi Jinping systematically expounded the ‘new normal’ for the first time*. *http*:*//www*.*xinhuanet*.*com//world/2014-11/09/c_1113175964*.*htm* *2014-11-09*). The efficiency of tourism development in other stages shows an upward trend. This finding is a direct reflection of GML index change.

Figs 6 and 7 show that GPTC and GSTC show an obvious upward trend in GML decomposition indexes. This finding indicates that the ‘fluctuated rising trend’ of GML indexes of tourism development of China’s major cities is mainly due to the technical progress of urban development in 2002–2016. In this paper, 58 major cities in China, excluding cities with good tourism resource endowment, are also the innovative cities with the most active economic development. Urban tourism development is represented by e-commerce, standardisation construction and product innovation. Tourism has high technological content as a whole and high utilisation ability of tourism resources. Furthermore, tourism resources can be allocated rationally. Therefore, the overall technical efficiency of urban tourism development is relatively high. However, the overall levels of GPTC and GSTC remain low currently. Promoting the overall technological progress of urban tourism development is necessary in the future by ways such as further strengthening technological innovation and developing tourism information technology.

GPEC and GSEC show a ‘fluctuated declining’ trend, reflecting that the overall level of haze governance in 58 cities has decreased during the period from 2001 to 2016. The Chinese government has increasingly advocated the concept of green development of the ecological economy. However, the industrial development in China’s cities remains highly dependent on resources and energy input at the current stage, so urban environmental problems remain prominent, especially haze. In addition, the market failure in the field of tourism economy leads to insufficient investment and repeated construction, making urban tourism development lose its features. The failure will also promote the environmental efficiency of urban tourism development to decline. Therefore, we should not only focus on the investment in environmental governance but also rationally guide the management and distribution of internal elements in the tourism system and fully use production factors in the development of urban tourism to improve the overall environmental efficiency of urban tourism development.

## 5 Conclusions and inspirations

In this paper, we analyse the efficiency of tourism development of 58 major cities in China under the constraint of haze characterised by PM2.5 emission concentration in 2001–2016. Among them, the input indicators to measure the efficiency of urban tourism development mainly include investment in fixed assets, employees in the tertiary industry and foreign direct investment. The output indicators mainly include total tourism income and total tourism population, and the PM2.5 emission concentration is used as an undesirable output. Furthermore, the EBM Super Efficiency Model is used to measure the efficiency of urban tourism development under the constraint of haze and the GML index is adopted to measure total factor productivity of tourism development in 58 cities in 2002–2016. These metrics are analysed through decomposing them into GPTC, GSEC, GPEC and GSTC. The main conclusions are as follows.

1. In 2001–2016, the efficiency of tourism development in 58 cities has been steadily improved. In the past 16 years, the average efficiency of urban tourism development has increased from 0.12 to 0.47. The resources input by the government for the city have an increasing effect on tourism development, but the overall efficiency is not high.
2. The efficiency of tourism development in all cities studied shows a fluctuated upward trend. Under the constraint of haze, the efficiency of urban tourism development is not directly proportional to the development of the city. In most western cities with less haze weather and good ecological environment, the efficiency of urban tourism development is higher than that in the eastern, central and northeastern cities instead.
3. We analyse the efficiency of urban tourism development from the perspective of input–output redundancy. In terms of all input redundancy improvement in 2001–2016, from the angle of input indicators, the overall redundancy rates in all input indicators of 58 major cities are slightly high, and the redundancy rates of input indicators in most cities are more than 50%. From the angle of output indicators, the output deficiency (redundancy) rates in all cities are greatly different. These findings are related to the resource endowment of different cities. In addition, compared with the redundancy rate of input indicators, output deficiency (redundancy) rate is generally higher. In the regions where 58 cities are located in 2001–2016, little difference occurs in input redundancy rate and output deficiency (redundancy) rate between the northeastern and western regions, but the redundancy rates of the two regions are far higher than those of the central and the eastern regions. Furthermore, the input redundancy rate and output deficiency (redundancy) rates of urban tourism development in the eastern region are much higher than those in the central region.
4. Under the constraint of haze, total factor productivity of tourism development in 58 cities shows a fluctuated upward trend in 2002–2016. The decomposition factors, GPTC and GSTC show a clear upward trend, and GPEC and GSEC show a fluctuated downward trend.

Based on the above conclusions, the following policy implications are drawn in this paper. Firstly, we should fully consider the differences in resource endowment, industrial structure characteristics and economic development stages of each city; formulate institutional arrangements to promote urban tourism development according to local conditions and strengthen the investment in soft resources such as modern information technology, service ability of employees and tourism commodity supply. Endogenous power can thus be provided to promote the efficiency of urban tourism development. Secondly, focusing on the governance of polluted weather; accelerating the relevant legislation; strengthening the education in hazards, causes and prevention of haze weather; improving the social responsibility of enterprises and individuals and advocating healthy production and lifestyle are necessary to reduce the emission of air pollutants and the occurrence of urban haze weather. Thirdly, we should deepen the supply-side structural reform of urban tourism development, cultivate the constraint mechanism of investment in science and technology and the dynamic mechanism of technological innovation, strengthen the supervision and management of resource elements and optimise the input structure to improve the performance of various resources in urban tourism development. Fourthly, taking sustainable development as guidance, we should guide urban tourism development through adhering to the concepts of Innovation, Coordination, Green, Opening and Sharing, as well as inhibit the restrictive effect of pollution emission and other environmental factors on the high-quality development of urban tourism by strengthening the supervision and management of emission reduction policies. Thus, a good ecological environment for the development of urban tourism can be created. In addition, we should scientifically grasp the internal driving factors of the total factor growth in urban tourism development, strengthen the collaborative innovation of science and technology and the construction of talent system and promote the high-quality development of urban tourism with the help of tourism technology progress, product and service innovation.

## References

[1] Bai X, Shi P, Liu Y. Realizing China’s urban dream. Nature. 2014;509: 158–160. doi: 10.1038/509158a 24812683

[2] Lu H, Yue A, Chen H, Long R. Could smog pollution lead to the migration of local skilled workers? Evidence from the Jing-Jin-Ji region in China. Resources, conservation and recycling. 2018;130: 177–187. doi: 10.1016/j.resconrec.2017.11.024

[3] Zhang X, Zhang X, Chen X. Happiness in the air: How does a dirty sky affect mental health and subjective well-being? Journal of Environmental Economics and Management. 2017. pp. 81–94. doi: 10.1016/j.jeem.2017.04.001 29081551 PMC5654562

[4] Chen Q, Zhang J, Xu Y, Sun H, Ding Z. Associations between individual perceptions of PM(2.5)pollution and pulmonary function in Chinese middle-aged and elderly residents. BMC Public Health. 2020;20. doi: 10.1186/s12889-020-08713-6 32522184 PMC7288539

[5] Wu K, Chen Y, Ma J, Bai S, Tang X. Traffic and emissions impact of congestion charging in the central Beijing urban area: A simulation analysis. Transportation Research Part D: Transport and Environment. 2017;51: 203–215. doi: 10.1016/j.trd.2016.06.005

[6] Zhang K, Hou Y, Li G, Huang Y. Tourists and Air Pollution: How and Why Air Pollution Magnifies Tourists’ Suspicion of Service Providers. Journal of Travel Research. 2020;59: 661–673. doi: 10.1177/0047287519859710

[7] Becken S, Jin X, Zhang C, Gao J. Urban air pollution in China: destination image and risk perceptions. Journal of Sustainable Tourism. 2017;25: 130–147. doi: 10.1080/09669582.2016.1177067

[8] Xu X, Dong D, Wang Y, Wang S. The Impacts of Different Air Pollutants on Domestic and Inbound Tourism in China. International Journal of Environmental Research and Public Health. 2019;16. doi: 10.3390/ijerph16245127 31847502 PMC6950462

[9] Russo MA, Relvas H, Gama C, Lopes M, Borrego C, Rodrigues V, et al. Estimating emissions from tourism activities. Atmospheric Environment. 2020;220: 117048. doi: 10.1016/j.atmosenv.2019.117048

[10] Xie H, Wang W. Spatiotemporal differences and convergence of urban industrial land use efficiency for China’s major economic zones. Journal of Geographical Sciences. 2015;25: 1183–1198. doi: 10.1007/s11442-015-1227-2

[11] Mullins P. Tourism Urbanization. International Journal of Urban and Regional Research. 1991;15: 326–342. doi: 10.1111/j.1468-2427.1991.tb00642.x

[12] Hetherington K. Acknowledging Consumption: A Review of New Studies. Economic Geography. 1997;73: 366–368. Available: https://search.proquest.com/docview/235658968?accountid=28839

[13] Hoffman L, Fainstein SS, Dennis R. Judd (eds.). Cities and Visitors: Regulating People, Markets, and City Space. Malden, MA: Blackwell Publishing Ltd.; 2003.

[14] Law CM. Urban Tourism and its Contribution to Economic Regeneration. Urban Studies. 1992;29: 599–618. doi: 10.1080/00420989220080581

[15] Edwards D, Griffin T, Hayllar B. Urban Tourism Research. Developing an Agenda. Annals of Tourism Research. 2008;35: 1032–1052. doi: 10.1016/j.annals.2008.09.002

[16] Robinson RNS. The Tourist Gaze 3.0. International Journal of Contemporary Hospitality Management. 2014;26: 154–156. doi: 10.1108/IJCHM-02-2013-0097

[17] Buckley R. Tourism and Environment. In: Gadgil, A and Liverman D, editor. Annual Review of Environment and Resources. 4139 EL CAMINO WAY, PO BOX 10139, PALO ALTO, CA 94303–0897 USA: ANNUAL REVIEWS; 2011. pp. 397–416. doi: 10.1146/annurev-environ-041210-132637

[18] Zhang D, Zhou C, Xu W. Spatial-Temporal Characteristics of Primary and Secondary Educational Resources for Relocated Children of Migrant Workers: The Case of Liaoning Province. Complexity. 2020;2020: 1–13. doi: 10.1155/2020/7457109

[19] Yang J, Sun J, Ge Q, Li X. Assessing the impacts of urbanization-associated green space on urban land surface temperature: A case study of Dalian, China. Urban Forestry & Urban Greening. 2017;22: 1–10. 10.1016/j.ufug.2017.01.002

[20] Nam PP, Yen TT. Impact of urbanization on land complaints in Vinh City, Nghe An province. Land Use Policy. 2021;108: 105533. 10.1016/j.landusepol.2021.105533

[21] Lai Z, Ge D, Xia H, Yue Y, Wang Z. Coupling coordination between environment, economy and tourism: A case study of China. Plos One. 2020;15. doi: 10.1371/journal.pone.0228426 32017789 PMC6999866

[22] Day J, Cai L. Environmental and energy-related challenges to sustainable tourism in the United States and China. International Journal of Sustainable Development and World Ecology. 2012;19: 379–388. doi: 10.1080/13504509.2012.675600

[23] Gkoumas A. Evaluating a standard for sustainable tourism through the lenses of local industry. Heliyon. 2019;5. doi: 10.1016/j.heliyon.2019.e02707 31840122 PMC6893070

[24] Buckley R. 20 Answers: Reconciling Air Travel and Climate Change. Annals of Tourism Research. 2011;38: 1178–1181. doi: 10.1016/j.annals.2011.03.019

[25] Weaver D. Can sustainable tourism survive climate change? Journal of Sustainable Tourism. 2010;19: 5–15. doi: 10.1080/09669582.2010.536242

[26] Santus P, Russo A, Madonini E, Allegra L, Blasi F, Centanni S, et al. How air pollution influences clinical management of respiratory diseases. A case-crossover study in Milan. Respiratory Research. 2012;13: 95. doi: 10.1186/1465-9921-13-95 23078274 PMC3511062

[27] Ruchiraset A, Tantrakarnapa K. Association of climate factors and air pollutants with pneumonia incidence in Lampang province, Thailand: findings from a 12-year longitudinal study. International Journal of Environmental Research. 2020;123: 10. doi: 10.1080/09603123.2020.1793919 32662678

[28] Sun XW, Chen PL, Ren L, Lin YN, Zhou JP, Ni L, et al. The cumulative effect of air pollutants on the acute exacerbation of COPD in Shanghai, China. Science of the total environment. 2018;622: 875–881. doi: 10.1016/j.scitotenv.2017.12.042 29227938

[29] Endler C, Matzarakis A. Analysis of high-resolution simulations for the Black Forest region from a point of view of tourism climatology—a comparison between two regional climate models (REMO and CLM). Theoretical and Applied Climatology. 2011;103: 427–440. doi: 10.1007/s00704-010-0311-x

[30] Matzarakis A, Haemmerle M, Koch E, Rudel E. The climate tourism potential of Alpine destinations using the example of Sonnblick, Rauris and Salzburg. Theoretical and Applied Climatology. 2012;110: 645–658. doi: 10.1007/s00704-012-0686-y

[31] Jinhua T, Jing S. Impact of intermittent vehicle release on freeway energy dissipation and emissions. Journal of Tsinghua University: Science and Technology. 2013;53: 499–502,508.

[32] Zolali M, Mirbaha B. Analysing the effect of foggy weather on drivers’ speed choice in two-lane highways. Proceedings of the Instittion of Civil Engineers-Transport. 2020;173: 171–183. doi: 10.1680/jtran.17.00140

[33] Zhou T, Zhang J. Analysis of commercial truck drivers’ potentially dangerous driving behaviors based on 11-month digital tachograph data and multilevel modeling approach. Accident Analysis and Prevention. 2019;132: 105256.1–105256.11. doi: 10.1016/j.aap.2019.105256 31442922

[34] Jin S, Yang J, Wang E, Liu J. The influence of high-speed rail on ice-snow tourism in northeastern China. Tourism Management. 2020;78. doi: 10.1016/j.tourman.2019.104055 32287754 PMC7126699

[35] Martin HS, del Bosque IAR. Exploring the cognitive-affective nature of destination image and the role of psychological factors in its formation. Tourism Management. 2008;29: 263–277. doi: 10.1016/j.tourman.2007.03.012

[36] Mainolfi G, Marino V. Destination beliefs, event satisfaction and post-visit product receptivity in event marketing. Results from a tourism experience. Journal of Business Research. 2020;116: 699–710. doi: 10.1016/j.jbusres.2018.03.001

[37] Lee B, Lee C-K, Lee J. Dynamic Nature of Destination Image and Influence of Tourist Overall Satisfaction on Image Modification. Journal of Travel Research. 2014;53: 239–251. doi: 10.1177/0047287513496466

[38] Almeida-Garcia F, Domigunez-Azcue J, Mercade-Mele P, Perez-Tapia G. Can a destination really change its image? The roles of information sources, motivations, and visits. Tourism Management Perspectives. 2020;34: 100662. doi: 10.1016/j.tmp.2020.100662

[39] Lapko A, Panasiuk A, Strulak-Wojcikiewicz R, Landowski M. The State of Air Pollution as a Factor Determining the Assessment of a City’s Tourist Attractiveness-Based on the Opinions of Polish Respondents. Sustainability. 2020;12: 1466. doi: 10.3390/su12041466

[40] Ruan W, Kang S, Song H. Applying protection motivation theory to understand international tourists’ behavioural intentions under the threat of air pollution: A case of Beijing, China. Current Issues in Tourism. 2020;23: 2027–2041. doi: 10.1080/13683500.2020.1743242

[41] Maha L-G, Viorica ED, Asandului M, Maha A. Hotel Efficiency Analysis from the Customer’s Point of View in Romania: A Stochastic Production Frontier Approach. Emerging Markets Finance and Trade. 2018;54: 661–676. doi: 10.1080/1540496X.2017.1421168

[42] Assaf AG, Magnini V. Accounting for customer satisfaction in measuring hotel efficiency: Evidence from the US hotel industry. International Journal of Hospitality Management. 2012;31: 642–647. doi: 10.1016/j.ijhm.2011.08.008

[43] Assaf AG, Atkinson SE, Tsionas MG. Endogeneity in multiple output production: Evidence from the US hotel industry. Tourism Management. 2020;80: 104–124. doi: 10.1016/j.tourman.2020.104124

[44] Angel Fernandez M, Becerra R. An Analysis of Spanish Hotel Efficiency. Cornell Hospitality Quarterly. 2015;56: 248–257. doi: 10.1177/1938965513509877

[45] Assaf AG, Tsionas M. Measuring hotel performance: Toward more rigorous evidence in both scope and methods. Tourism Management. 2018;69: 69–87. doi: 10.1016/j.tourman.2018.05.008

[46] Koksal CD, Aksu AA. Efficiency evaluation of A-group travel agencies with data envelopment analysis (DEA): A case study in the Antalya region, Turkey. Tourism Management. 2007;28: 830–834. doi: 10.1016/j.tourman.2006.05.013

[47] Dragan D, Keshavarzsaleh A, Jereb B, Topolsek D. Integration with transport suppliers and efficiency of travel agencies. International Journal of Value Chain Management. 2018;9: 122–148. doi: 10.1504/IJVCM.2018.092388

[48] Ramirez-Hurtado JM, Contreras I. Efficiency of travel agency franchises: a study in Spain. Service Business. 2017;11: 717–739. doi: 10.1007/s11628-016-0326-1

[49] Mahmoudi R, Emrouznejad A, Shetab-Boushehri S-N, Hejazi SR. The origins, development and future directions of data envelopment analysis approach in transportation systems. Socio-Economic Planning Sciences. 2020;69: 1–15. doi: 10.1016/j.seps.2018.11.009

[50] Zha J, He L, Liu Y, Shao Y. Evaluation on development efficiency of low-carbon tourism economy: A case study of Hubei Province, China. Socio-Economic Planning Sciences. 2019;66: 47–57. doi: 10.1016/j.seps.2018.07.003

[51] Charnes A, Cooper WW, Rhodes E. Measuring the efficiency of decision making units. European Journal of Operational Research. 1978;2: 429–444. doi: 10.1016/0377-2217(78)90138-8

[52] Dyckhoff H, Allen K. Measuring ecological efficiency with data envelopment analysis (DEA). European Journal of Operational Research. 2001;132: 312–325. doi: 10.1016/S0377-2217(00)00154-5

[53] Qin Q, Li X, Li L, Zhen W, Wei YM. Air emissions perspective on energy efficiency: An empirical analysis of China’s coastal areas. Applied Energy. 2017;185: 604–614. doi: 10.1016/j.apenergy.2016.10.127

[54] Pittman RW. Issue in Pollution Control: Interplant Cost Differences and Economies of Scale. Land Economics. 1981;57: 1–17. doi: 10.2307/3145748

[55] Reinhard S, Knox Lovell CA, Thijssen GJ. Environmental efficiency with multiple environmentally detrimental variables; estimated with SFA and DEA. European Journal of Operational Research. 2000;121: 287–303. 10.1016/S0377-2217(99)00218-0

[56] Hailu A, Veeman TS. Non-parametric Productivity Analysis with Undesirable Outputs: An Application to the Canadian Pulp and Paper Industry. American Journal of Agricultural Economics. 2001;83: 605–616. doi: 10.1111/0002-9092.00181

[57] Liu WB, Meng W, Li XX, Zhang DQ. DEA models with undesirable inputs and outputs. Annals of Operations Research. 2010;173: 177–194. doi: 10.1007/s10479-009-0587-3

[58] Tone K, Sahoo BK. Degree of scale economies and congestion: A unified DEA approach. European Journal of Operational Research. 2004;158: 755–772. 10.1016/S0377-2217(03)00370-9

[59] Zhang J, Zeng W, Shi H. Regional environmental efficiency in China: Analysis based on a regional slack-based measure with environmental undesirable outputs. Ecological Indicators. 2016;71: 218–228. 10.1016/j.ecolind.2016.04.040

[60] Tone K, Tsutsui M. An epsilon-based measure of efficiency in DEA–A third pole of technical efficiency. European Journal of Operational Research. 2010;207: 1554–1563. doi: 10.1016/j.ejor.2010.07.014

[61] Van Donkelaar A, Martin R V., Brauer M, Boys BL. Use of satellite observations for long-term exposure assessment of global concentrations of fine particulate matter. Environmental Health Perspectives. 2015. pp. 135–143. doi: 10.1289/ehp.1408646 25343779 PMC4314252

[62] Houkai W, Yeqiang W, Hongjian S, Yebo G. Comprehensive Evaluation Report on China’s Urbanization Quality. Review of Economic Research. 2013;31: 3–32. doi: 10.16110/j.cnki.issn2095-3151.2013.31.002

[63] Byrnes PE, Storbeck JE. Efficiency gains from regionalization: economic development in China revisited. Socio-Economic Planning Sciences. 2000;34: 141–154. 10.1016/S0038-0121(99)00022-1

[64] Yongan D. The Efficiency and Determinants of China’s Urbanization. The Journal of Quantitative & Technical Economics. 2010;12.

[65] Halleux J-M, Marcinczak S, van der Krabben E. The adaptive efficiency of land use planning measured by the control of urban sprawl. The cases of the Netherlands, Belgium and Poland. Land Use Policy. 2012;29: 887–898. 10.1016/j.landusepol.2012.01.008

[66] Zhou C, Shi C, Wang S, Zhang G. Estimation of eco-efficiency and its influencing factors in Guangdong province based on Super-SBM and panel regression models. Ecological Indicators. 2018;86: 67–80. doi: 10.1016/j.ecolind.2017.12.011

[67] Kaiming C, Cunzhang D. Correlative mechanism and dynamic econometric analysis between FDI and urbanization in china. Economic Geography. 2010;30: 99–103. doi: 10.1017/CBO9781107415324.004

[68] Wu W, Zhao K. Dynamic interaction between foreign direct investment and the new urbanization in China. Journal of Housing and the Built Environment. 2019. pp. 1107–1124. doi: 10.1007/s10901-019-09666-y

[69] Ma X-L. Evaluation of Tourism Total Factor Productivity for Chinese Primary Cities from 2000 to 2011. Resources Science. 2014;36: 1626–1634.

[70] Huang CW, Chen HY, Ting C Te. Using a network data envelopment analysis model to assess the efficiency and effectiveness of cultural tourism promotion in Taiwan. Journal of Travel and Tourism Marketing. 2017;34: 1274–1284. doi: 10.1080/10548408.2017.1345342

[71] Ma X-L, Ryan C, Bao J-G. Chinese national parks: Differences, resource use and tourism product portfolios. Tourism Management. 2009;30: 21–30. doi: 10.1016/j.tourman.2008.04.006

[72] Yi T, Liang M. Evolutional Model of Tourism Efficiency Based on the DEA Method: A Case Study of Cities in Guangdong Province, China. Asia Pacific Journal of Tourism Research. 2015;20: 789–806. doi: 10.1080/10941665.2014.932294

[73] Cao F, Huang Z, Jin C, Xu M. Influence of Chinese economic fluctuations on tourism efficiency in national scenic areas. Tourism Economics. 2016;22: 884–907. doi: 10.5367/te.2015.0463

[74] Peng H, Zhang J, Lu L, Tang G, Yan B, Xiao X, et al. Eco-efficiency and its determinants at a tourism destination: A case study of Huangshan National Park, China. Tourism Management. 2017;60: 201–211. doi: 10.1016/j.tourman.2016.12.005

[75] Wang E, Wu D. A Study on Spatial-Temporal Differences of Tourism Urbanization Efficiency of Tourism-driven City. Social Sciences in Nanjing. 2016; 29–35.

[76] Shao S. Urbanization Promotion and Haze Pollution Governance in China. Economic Research Journal. 2019; 148–165.

[77] Wei W, Weiping D. Three-stage Innovation Efficiency of High-tech Industry and Its Influencing Factors——Based on EBM Model and Tobit Model. Soft Science. 2017;31: 16–20.

[78] Banker RD, Charnes A, Cooper WW. Some Models for Estimating Technical and Scale Inefficiencies in Data Envelopment Analysis. Management Science. 1984. pp. 1078–1092. doi: 10.1287/mnsc.30.9.1078

[79] Tone K. A slacks-based measure of efficiency in data envelopment analysis. European Journal of Operational Research. 2001;130: 498–509. 10.1016/S0377-2217(99)00407-5

[80] Li L, Liu B. Efficiency Evaluation and Causes Identification of Chinese High-tech Industry. Economic Perspectives. 2014; 56–65.

[81] Gang C. Data Envelopment Analysis Method and MAXDEA Software. BeiJing: Intellectual Property Publishing House; 2014.

[82] Andersen P, Petersen NC. A Procedure for Ranking Efficient Units in Data Envelopment Analysis. Management Science. 1993;39: 1261–1264. doi: 10.1287/mnsc.39.10.1261

[83] Chung YH, Färe R, Grosskopf S. Productivity and Undesirable Outputs: A Directional Distance Function Approach. Journal of Environmental Management. 1997;51: 229–240. 10.1006/jema.1997.0146

[84] Liangshi Z, Caizhi S. Water Resource Total Factor Productivity Efficiency in China Using the Global-Malmquist-Luenberger Index. Resources Science. 2013;35: 1229–1237.

[85] Yawei Q. Measurement and Decomposition of China’s Total Factor Productivity Under the Constraints of Carbon Emissions. Industrial Technology & Economy. 2013; 137–146.

[86] Oh D, Lee J. A metafrontier approach for measuring Malmquist productivity index. Empirical Economics. 2010;38: 47–64. doi: 10.1007/s00181-009-0255-0

[87] Zofio JL. Malmquist productivity index decompositions: a unifying framework. Applied Economics. 2007;39: 2371–2387. doi: 10.1080/00036840600606260

[88] Fare R, Grosskopf S, Norris M, Zhongyang Zhang. Productivity growth, technical progress, and efficiency change in industrialized countries. American Economic Review. 1994;84: 66–83. doi: 10.2307/2117971

